# Increased Soluble Urokinase-Type Plasminogen Activator Receptor (suPAR) Levels in Plasma of Suicide Attempters

**DOI:** 10.1371/journal.pone.0140052

**Published:** 2015-10-09

**Authors:** Filip Ventorp, Anna Gustafsson, Lil Träskman-Bendz, Åsa Westrin, Lennart Ljunggren

**Affiliations:** 1 Division of Psychiatry, Department of Clinical Sciences, Lund University, Lund, Sweden; 2 Department of Biomedical Sciences, Malmö University, Malmö, Sweden; Shinshu University School of Medicine, JAPAN

## Abstract

The soluble form of the urokinase receptor, suPAR, has been suggested as a novel biomarker of low-grade inflammation. Activation of the immune system has been proposed to contribute to the development of depression and suicidal behavior. In order to identify depressed and suicidal individuals who could benefit from an anti-inflammatory treatment, a reliable biomarker of low-grade inflammation is vital. This study evaluates plasma suPAR levels as a biomarker of low-grade inflammation in patients with major depressive disorder and in patients who recently attempted suicide. The plasma suPAR and an established biomarker, C reactive protein (CRP) of suicide attempters (n = 54), depressed patients (n = 19) and healthy controls (n = 19) was analyzed with enzyme-linked immunosorbent assays. The biomarker attributes of sensitivity and sensibility were evaluated using ROC curve analysis. Both the depressed patients and suicide attempters had increased plasma suPAR. The levels of suPAR discriminated better between controls and suicide attempters than did CRP. In the future, plasma suPAR might be a superior prognosticator regarding outcome of treatment applying conventional antidepressants in conjunction with anti-inflammatory drugs.

## Introduction

Major depressive disorder (MDD) is characterized by low mood, anhedonia and reduced energy [1]. It can cause long-term disability and is a source of significant public health costs [2, 3]. It is also a disorder associated with a high mortality rate since suicide ends the life of about 4% of patients with MDD [4]. The main mechanism of action of commonly used antidepressants is to increase synaptic levels of one or more monoamines e.g. serotonin, noradrenalin, and dopamine [5]. However, neurotransmitter reuptake inhibitors lead to remission only in about 30–45% of patients [6, 7], hence other factors than brain monoamines might be important in the pathogenesis of depression.

Activation of the immune system has been suggested to contribute to the development of depression [8, 9]. Patients diagnosed with MDD exhibit increased levels of some cytokines in the blood [10]. Moreover, a significant number of patients treated with the immune-activating drug IFN-alpha, as a therapy against hepatitis C or certain cancers, also develop a depressed mood [11]. There are also studies linking inflammation with suicidality [12]. Post-mortem examinations of the brain from suicide victims have shown high transcript levels of interleukin-4 and interleukin-13 in the orbitofrontal cortical area [13] and microgliosis in the dorsolateral prefrontal cortex and anterior cingulate cortex [14]. Suicide attempters have also been reported to have higher levels of interleukin-6 (IL-6) in the cerebrospinal fluid (CSF) and higher levels of IL-6 and the tumor necrosis factor alpha (TNF-alpha) in plasma compared to healthy controls [15, 16].

Even though inflammatory processes appear to contribute to the development of depression in some patients; not everyone with depression has signs of inflammation [17]. In the subgroup of psychiatric patients who show signs of inflammation, an anti-inflammatory treatment might be beneficial. Interestingly, an add-on treatment with celecoxib (NSAID) to a reboxetine treatment (norepinephrine reuptake inhibitor, NRI), has been shown to have a therapeutic effect in MDD [18]. In a second study, the proinflammatory activity in patients predicted the antidepressant response to the celecoxib therapy, i.e. only the patients with some degree of pro-inflammatory activity, responded to an add-on treatment with celecoxib [19]. In a clinical perspective, to find candidates for an anti-inflammatory treatment, it is vital to distinguish depressive patients with an ongoing low-grade inflammation from depressive patients without inflammation. Therefore, a biomarker of low-grade inflammation in patients with an MDD diagnosis is imperative.

The urokinase receptor, uPAR, is part of the plasminogen activation system. uPAR is also involved in cellular adhesion and migration and is important for the recruitment of immune cells [20, 21]. The soluble form of the receptor, suPAR, results from the cleavage and release of membrane-bound uPAR into the blood and reflects the activation of the immune system. In most cases serum levels of suPAR positively correlate with inflammatory proteins such as TNF-alpha, and CRP [22, 23]. In addition to plasma, suPAR is found in various other body fluids, such as urine and CSF [24]. In contrast to cytokines [25, 26], suPAR is not further degraded and can therefore be assayed in various body fluids. Furthermore, suPAR levels are stable throughout the day in plasma, independent of the circadian rhythm or if the subject is fasting or not. Repeated freeze-thaw procedures of samples do not affect the concentrations. Compared to CRP and IL-6, suPAR levels are not strongly linked to anthropometric measures such as body mass index (BMI) and waist circumference [27]. Hence, suPAR holds promise as a reliable biomarker of low-grade inflammation [24]. Recently, high suPAR levels were associated with an increased likelihood for a diagnosis of depression [28].

In this study, we ventured to evaluate suPAR as a biomarker of low-grade inflammation in patients with MDD and in patients who recently attempted suicide. We hypothesized that suPAR would be better to identify psychiatric patients with low-grade inflammation than the conventional biomarker CRP.

## Methods

### Study Participants

All patients gave written informed consent to participate and the Lund University Medical Ethics Committee approved the studies. The depressed patients (n = 19) were recruited from the psychiatric clinic at Lund University Hospital. An inclusion criterion was a DSM-IV diagnosis of moderate to severe MDD. Exclusion criteria were pregnancy, cardiovascular disease, and treatment with antidepressants, neuroleptics, or mood stabilizers during the last month. Suicide attempters (n = 54) were enrolled following admission to Lund University Hospital after a suicide attempt. The control group (n = 19) was randomly selected from the municipal population register in Lund, Sweden. Blood samples from healthy controls and non-suicidal depressed patients were collected between 2001–2003 and from suicide attempters between 2006–2008. The inclusion criteria for control subjects were good physical health, no history of current mental or somatic disorder. The demographic data of depressed patients, suicide attempters and controls are listed in Table 1. Some depressed patients and suicide attempters had an ongoing somatic condition; these conditions are listed also in Table 2.

**Table 1:** The demographic data of the study participants.

	Controls	Depressed Patients	Suicide Attempters
**Sex (male/female) male %**	(9 / 10) 47.4%	(9 / 10) 47.4%	(24 / 30) 44.4%
**Age ± SD**	34.7 ± 10.8	34.0 ± 10.3	38.5 ± 14.5
**BMI ± SD**	23.3 ± 3.1	24.9 ± 7.6	25.7 ± 4.4

**Table 2:** Somatic diagnoses of the suicide attempters and non-suicidal depressed patients.

Somatic conditions		
***Diagnosis***	*Suicide Attempters*	*Non-suicidal depressed patients*
Endocrine-nutritional and metabolic diseases	7	2
Nervous system diseases	4	2
Eye diseases	1	–
Ear diseases	1	–
Circulatory system diseases	3	–
Respiratory tract diseases	1	1
Digestive system diseases	1	1
Skin diseases	–	1
Musculoskeletal diseases	2	–
Pain conditions	3	–
Allergy	2	–

Axis I diagnoses of the suicide attempters were set at an interview by a specialist in psychiatry, according to DSM-IV. The patients were diagnosed as: schizoaffective disorder (*n* = 2), psychotic disorder NOS (*n* = 1), major depressive disorder (*n* = 12), bipolar I disorder (*n* = 3), bipolar II disorder (*n* = 12), anxiety disorder NOS (*n* = 4), generalized anxiety disorder (*n* = 1), dysthymic disorder (*n* = 3), alcohol dependence (*n* = 6), substance dependence (*n* = 3), adjustment disorder (*n* = 3), adjustment disorder with depressed mood (*n* = 1) and depressive disorder NOS (*n* = 3).

Suicide attempters had the following psychiatric medications: SNRI (*n* = 11), SSRI (*n* = 10), Neuroleptics + SSRI (*n* = 6), Anti-epileptics (*n* = 6) Anti-epileptics + other (*n* = 4), Lithium (*n* = 3) and other combinations (*n* = 9).

### Sample handling

Blood samples were taken with EDTA coated vacutainers. The samples were immediately placed on ice and centrifuged at 4°C and 3000 rpm for 10 min within 1 h of collection. Plasma was stored at −80°C until further analysis.

### Analyte measurements

Plasma (EDTA) suPAR concentrations were analyzed using a commercially available enzyme immunoassay (suPARnostic™, Virogates, Copenhagen, Denmark), according to the manufacturer’s instructions. The assay detects all circulating suPAR, including intact and cleaved forms of the receptor. CRP was measured using a highly sensitive ELISA (Immundiagnostik AG, Bernsheim, Germany), according to the manufacturer’s instructions. Samples were analyzed in duplicates and the mean was used for statistical analysis. The concentration of analytes were above the detection limit in all samples. Detection limits: suPAR = 0.1 ng/ml, CRP = 0.9 ng/ml.

### Statistical Analysis

The statistical analysis was undertaken using SPSS Statistics 22 (IBM, Armonk, New York, US) and RKWard v0.60, R v 2.15.3 (rkward.sourceforge.net). The ROC curve graph was made by the R package ROCR v1.0–5 (rocr.bioinf.mpi-sb.mpg.de). Both suPAR and CPR correlated with age and BMI, p < 0.010. There was no significant correlation between any of the analytes and sex. Consequently, ANCOVA (GLM) was used to compare the mean values between healthy controls, depressed patients and suicide attempters, controlling for age and BMI when applicable. All results were additionally analyzed by non-parametric tests (without the possibility of adjustments). The variables were checked regarding assumptions of normality, homogeneity of variance, and homogeneity of regression slopes. Spearman’s rho was used for correlation analysis (besides analyzing effects of cofounders). The alpha-level of significance was set at p = 0.05. A SPSS file with data used in the statistical analysis is included as a supplemental item (S1 Dataset).

## Results

### suPAR and CRP levels in depressed patients and suicide attempters

**Fig 1:**
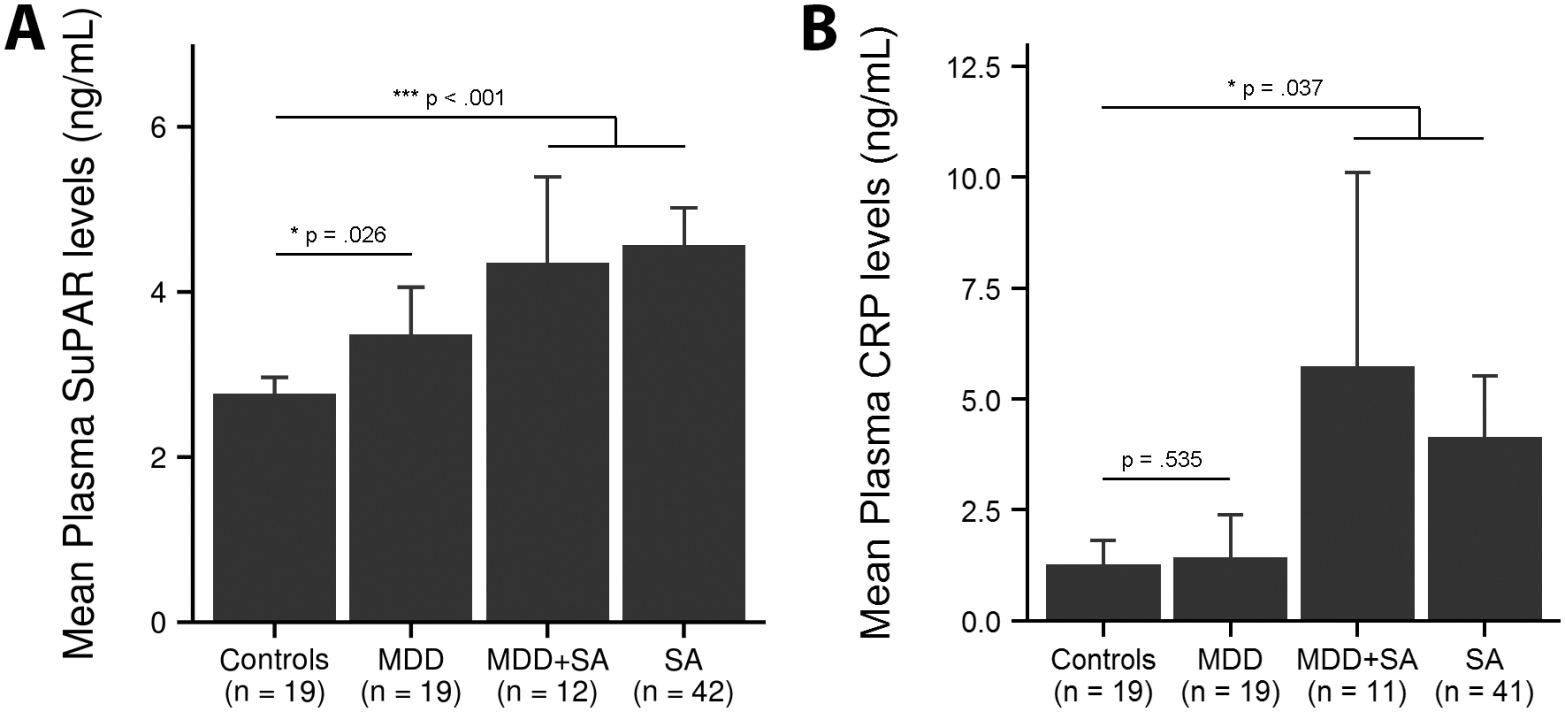
suPAR and CRP levels in depressed patients and suicide attempters. **(A)** The plasma mean levels of suPAR in healthy controls, depressed patients and suicide attempters. Both depressed patients and suicide attempters had higher levels of suPAR than the controls. In the figure, the suicide attempters have been divided into two subgroups based on the axis 1 diagnoses; one subgroup with patients diagnosed with MDD and one subgroup with the remaining patients with various diagnoses. However, the suicide attempters were considered as belonging to one group in the statistical analysis. **(B)** The mean CRP levels in plasma in healthy controls, depressed patients and suicide attempters. Suicide attempters had significantly higher levels compared to healthy controls. ANCOVA was used for mean value analysis, adjusting for age and BMI. MDD = Major Depressive Disorder, SA = Suicide Attempters. Error bars 95% CI.

The levels of suPAR were significantly higher in suicide attempters (4.53 ± 1.51 ng/mL, mean ± SD, 58.2% higher, adjusted for the effect of age and BMI, n = 54) than in healthy controls (2.76 ± 0.42 ng/mL, n = 19), Fig 1A (ANCOVA, p < 0.001). These levels were also higher in depressed patients (3.49 ± 1.18 ng/mL compared to healthy controls (p = 0.026), the difference did however not reach statistical significance after corrections for multiple comparisons. There was no significant difference of suPAR between depressed patients and suicide attempters.

There were no significant differences in CRP levels between healthy controls and depressed patients (ANCOVA, NS), Fig 1B. However there was a trend of higher levels of CRP in suicide attempters (p = 0.037) but it was below the alpha-level of significance after corrections for multiple comparisons. CRP correlated significantly with suPAR (Spearman’s rho = 0.52, p < 0.001, n = 71 [controls excluded]).

### suPAR levels discriminate between healthy controls and suicide attempters better than CRP levels

**Fig 2:**
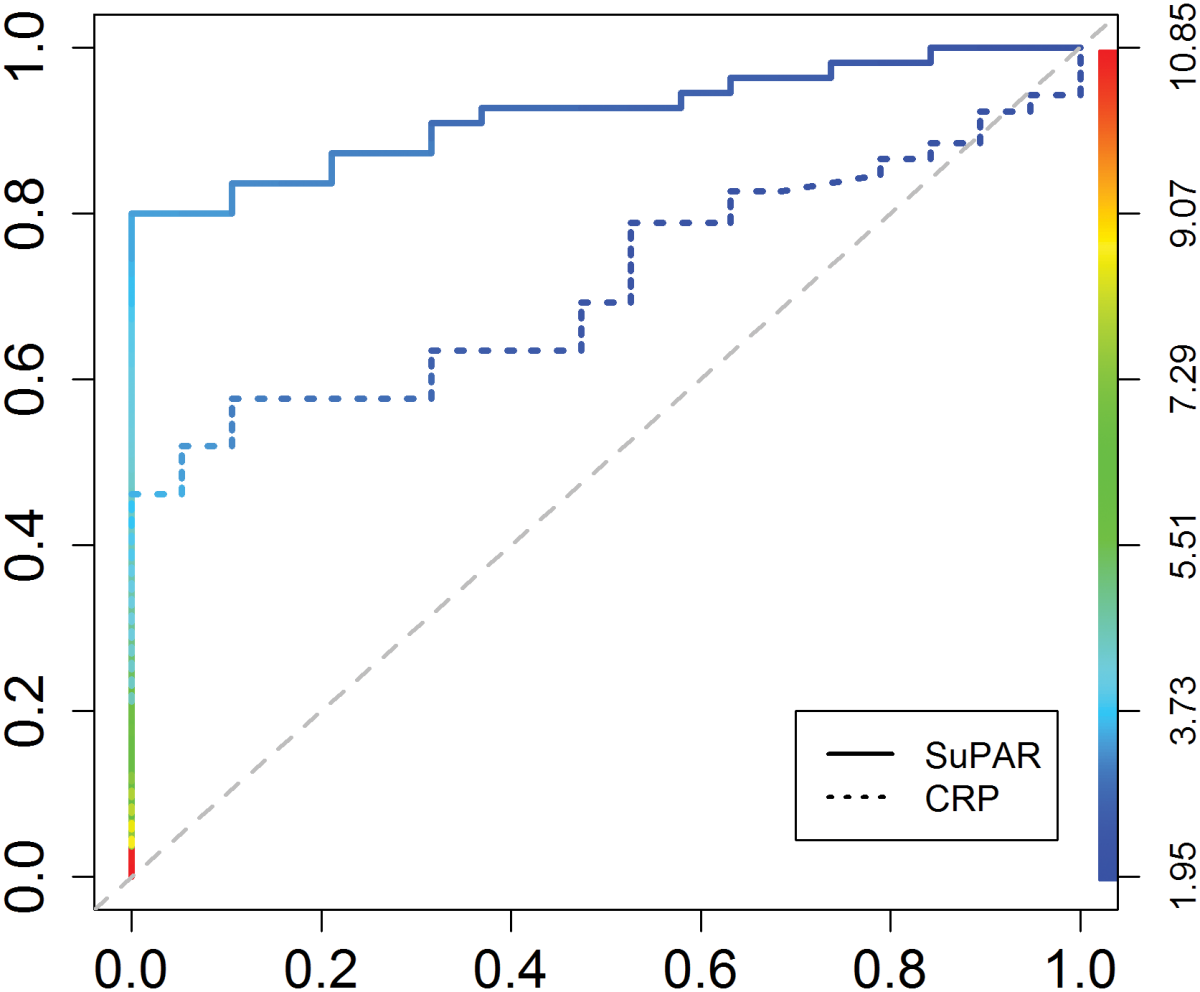
suPAR levels discriminates between healthy controls and suicide attempters better than CRP levels. ROC curve analysis of controls and suicide attempters as a state variable (the positive actual state is “suicide attempter”), and suPAR and CRP as classifiers. The color key on the right y-axis illustrates the spectrum of different cut-off values of suPAR levels. Left y-axis = true positive rate, x-axis = false positive rate. The area under the curve (AUC) for suPAR = 0.915 and CRP = 0.715. The significance of the difference between the areas below the curves of suPAR and CRP equals p = 0.0053.

To visualize and analyze the use of suPAR and CRP as a diagnostic test to discriminate between controls and suicide attempters, a ROC curve analysis was performed. suPAR plasma levels demonstrate a better classifier performance than CRP levels since the area under the curve (AUC) of suPAR levels are 0.915 compared to 0.715, see Fig 2. The significance of the difference between the areas below the curves of suPAR and CRP equals p = 0.0053.

## Discussion

Our results show that suPAR levels are increased in patients who recently made a suicide attempt. High levels of suPAR were also seen in a cohort of patients with MDD, but the levels and the statistical significance were not as distinctive as in the suicide attempters. However, the result supports a prior study that showed that elevated suPAR levels might reflect an increased risk of depression [28]. Increased levels of inflammatory markers, such as CRP, have previously been recorded both in depressed patients and in suicidal patients [10, 15, 16]. When we compared the mean values of suPAR levels with CRP in suicide attempters and controls, we saw a more pronounced difference in the suPAR levels. Furthermore, as a diagnostic test, suPAR compared to CRP shows better sensitivity and specificity in distinguishing healthy controls from suicide attempters.

Increased serum suPAR levels have been documented in several illnesses such as HIV-infection, malaria, sepsis, bacterial and viral CNS infection, and certain cancers. suPAR is considered a marker of inflammation and in this study suPAR levels were strongly associated with the levels of CRP. In studies linking low-grade inflammation to depression and suicidality, pro-inflammatory cytokines have usually been measured. However, the use of cytokines as a biomarker for low-grade inflammation has some disadvantages. Due to their local mode of action, involving paracrine or autocrine processes, cytokines circulate at very low levels (< 10 pg/ml), often below or near the detection limit of the majority of assays [29] which results in problems with assay precision. The majority of cytokines are sensitive to degradation. Plasma or serum samples have to be handled accordingly to improve stability, e.g. frozen rapidly after collection and thawing-freezing cycles should be avoided [29]. Since more and more research shows that cytokines are pleiotropic, there is also a problem of specificity. For example, the cytokine IL-6 is also considered to be a myokine and is elevated in blood in response to muscle contractions [30], and it has been estimated that 30% of the total circulating IL-6 originates from adipose tissue [27]. Furthermore, both the pro-inflammatory cytokines TNF-alpha, and IL-6 are in some settings considered to have anti-inflammatory properties [31, 32]. Many cytokines also follow a circadian rhythm which demand sample acquisition to take place at a standardized time point, most often in the morning [33]. There are also many other confounders, which could affect the levels of cytokines in the blood. For example, correlations with cytokines and BMI, age, smoking, sex, and physical activity have been reported [34, 35]. suPAR levels are independent of the circadian rhythm, not sensitive to repeated freeze-thaw [25, 26], and not strongly linked to BMI [27]. However, sex, age and current smoking has been reported to be associated with suPAR levels [36]. We found correlations between suPAR levels, age and BMI, but as a limitation of this study we were unable to adjust for current smoking.

Although our suicide attempt cohort has higher levels of suPAR than healthy controls, it could be suggested that this is the result of the suicide attempt itself, and not an indication of a low-grade inflammation in the subjects prior to the actual suicide attempt. The experimental design of this study cannot exclude the possibility that activation of inflammatory processes were not present before the suicide attempt, but there are some elements that make it unlikely. For example, the blood from the suicide attempters was collected approximately two weeks after the suicide attempt. Most likely, if any wounds were inflicted at the suicide attempt, they would have been healed at this time point. Furthermore, when dividing suicide attempts in groups based on the nature of the attempt, i.e. violent (e.g. hanging) and non-violent (intoxications) suicide attempts, there are no differences in suPAR levels between these two groups (data not shown) which implicates that suicide attempts likely to inflict injury did not affect the levels of suPAR in plasma. However, a study including a group of patients with suicide ideation, but without committed suicide attempts, is warranted to fully address this objection.

Recently, suPAR levels in blood from patients with schizophrenia were compared to healthy controls [36]. Interestingly, the mean values of suPAR levels were higher in patients with schizophrenia and the mean value of suPAR corresponded to the suPAR levels in suicide attempters of this study (i.e. approximately 4 ng/mL). In our cohort, only two suicide attempters had a concurrent schizoaffective disorder, making it impossible to investigate the separate effect of a schizophrenia diagnoses from the effect of suicide behavior and major depressive disorder on the levels of suPAR. However, just like suicide behavior and depression, the immune system has been implicated in the etiology of schizophrenia [37–39]. It is possibly, low grade inflammation does not specifically increase the risk of one psychiatric disorder, but rather several, in the same manner smoking increases the risk of not one but several different somatic diseases.

Even though there are studies showing that people with depression have elevated levels of inflammatory biomarkers, such as TNF-alpha, IL-6, and CRP, inflammation is not necessary or sufficient for the diagnosis of depression. Though the mean values of the inflammatory levels are significantly higher in patients, the levels of markers overlap to a great deal between the controls and the patients [17]. This is true for the suPAR and CRP levels in this study as well. Since MDD and suicidality are considered to have multifactorial etiologies, the overlap could be explained by the fact that only a subgroup of the patients have ongoing inflammatory processes that contribute to the pathogenesis of depression and suicidality. This hypothesis is strengthened by a study from Muller et al. showing that patients with elevated TNF-alpha levels in the blood responded better to treatment with conventional antidepressants plus an add-on treatment with an anti-inflammatory drug (i.e. NSAID) [18]. In the future, it could be important to find a biomarker, which can discriminate between depressed patients with a current inflammation and those without. Similarly to CRP, commercial quick-tests are available to measure suPAR fast in a clinical setting. Since suPAR is a reliable biomarker of low-grade inflammation and since we in the present study show that suPAR levels better discriminate between controls and patients than CRP, we conclude that suPAR is a promising alternative.

The samples from suicide attempters were collected approximately five years later than those from healthy controls and depressed non-suicidal patients. The difference in suPAR levels between healthy controls and suicide attempters might partly be explained by degradation of suPAR in the control samples due to longer duration of freezer storage. However, suPAR levels in plasma samples are known to be stable through repeated freezing/thawing [40]. Freezing storage do not appear to have a major influence on samples stored almost for one year [41]. On the other hand, long-term freezer storage leads to water evaporation resulting in increased protein levels, hence the actual differences between healthy controls and suicide attempters might be greater. There are some other limitations to our study. Some of the subjects in both the patients groups and controls had various somatic medical conditions. To strengthen the link between suPAR and specifically suicide behavior and depression, future studies should include subjects without somatic conditions and possibly medication free patients. The sample size in each group is small and should be viewed as preliminary, future studies replicating this finding is necessary.

In conclusion, we have shown that depressed patients and suicide attempters have increased plasma levels of suPAR. The levels of suPAR are better suited to discriminate between controls and suicide attempters than CRP levels. This study strengthens the hypothesis that a subgroup of depressed and suicide attempters have ongoing inflammatory processes. Future studies are warranted to investigate if a high suPAR levels is a good predictor of a better outcome of treatment using conventional antidepressants in conjunction with anti-inflammatory drugs.

## Supporting Information

S1 Dataset SPSS Statistics Data file. A SPSS file with data used in the statistical analysis. Covariates were excluded in the file due to restrictions of the ethical permission. However a complete file is provided for researchers after request at publication@ventorp.com.
(SAV)
